# Monitoring and modeling of household air quality related to use of different Cookfuels in Paraguay

**DOI:** 10.1111/ina.12513

**Published:** 2019-01-30

**Authors:** Matias Tagle, Ajay Pillarisetti, Maria Teresa Hernandez, Karin Troncoso, Agnes Soares, Ricardo Torres, Aida Galeano, Pedro Oyola, John Balmes, Kirk R. Smith

**Affiliations:** ^1^ Environmental Health Sciences School of Public Health University of California at Berkeley Berkeley California; ^2^ Centro Mario Molina Chile Providencia, Santiago Chile; ^3^ Pan American Health Organization Washington District of Columbia; ^4^ Dirección General de Salud Ambiental San Lorenzo Paraguay; ^5^ School of Medicine University of California San Francisco California

**Keywords:** biomass, CO, household air pollution, multiple linear regression, outdoor air pollution, PM_2.5_

## Abstract

In Paraguay, 49% of the population depends on biomass (wood and charcoal) for cooking. Residential biomass burning is a major source of fine particulate matter (PM_2.5_) and carbon monoxide (CO) in and around the household environment. In July 2016, cross‐sectional household air pollution sampling was conducted in 80 households in rural Paraguay. Time‐integrated samples (24 hours) of PM_2.5_ and continuous CO concentrations were measured in kitchens that used wood, charcoal, liquefied petroleum gas (LPG), or electricity to cook. Qualitative and quantitative household‐level variables were captured using questionnaires. The average PM_2.5_ concentration (μg/m^3^) was higher in kitchens that burned wood (741.7 ± 546.4) and charcoal (107.0 ± 68.6) than in kitchens where LPG (52.3 ± 18.9) or electricity (52.0 ± 14.8) was used. Likewise, the average CO concentration (ppm) was higher in kitchens that used wood (19.4 ± 12.6) and charcoal (7.6 ± 6.5) than in those that used LPG (0.5 ± 0.6) or electricity (0.4 ± 0.6). Multivariable linear regression was conducted to generate predictive models for indoor PM_2.5_ and CO concentrations (predicted *R* ^2^ = 0.837 and 0.822, respectively). This study provides baseline indoor air quality data for Paraguay and presents a multivariate statistical approach that could be used in future research and intervention programs.

1

**Practical Implications**

- Household air pollution associated with cooking fuels has been well documented in various developing countries but not in Paraguay.
- The paper reports the first indoor air quality monitoring campaign conducted in the country.
- These data could be used to model indoor air quality in similar settings and to develop national policies aiming to reduce exposure to household air pollution.

## 2 INTRODUCTION

Solid biomass, mainly wood and charcoal, accounts for approximately 43% of total annual energy consumption in Paraguay.^1^ These fuels are largely used by industry and the 49% of the population that depends on biomass for cooking.^2^

Concerns about biomass‐burning cookstoves have grown in recent decades, as their emissions are a significant source of climate‐altering pollutants and carbonaceous aerosols that contribute to the global burden of disease^3^, ^4^, ^5^. Emissions from biomass‐burning cookstoves contain high concentrations of health‐damaging products of incomplete combustion,^6^ such as carbon monoxide (CO) and PM_2.5_. The latter refers to particulate matter that has an aerodynamic diameter less than or equal to a nominal 2.5 micrometer (μm).

It has been documented that acute exposure to high concentrations of CO can cause death within minutes, while chronic low‐level exposure can lead to harmful neurological effects.^7^ The health issues of prolonged exposure to PM_2.5_ from cooking with biomass have been associated with a higher risk of suffering pneumonia in children and cardiovascular and pulmonary diseases in adults.^8^, ^9^

Biomass‐burning cookstoves have the potential to produce indoor air pollution when used in poorly ventilated household environments. As an environmental factor, household air pollution has been associated with an increased risk of premature death.^10^ Due to the threat it poses to public health, the World Health Organization (WHO) has established guidelines on indoor air quality exclusively related to household fuel combustion.^11^ These recommend that indoor PM_2.5_ concentration should not exceed 10 μg/m^3^ as an annual average, while the daily CO average should be below the threshold of 7 mg/m^3^, approximately 5.7 parts per million (ppm).

Despite WHO suggestions, healthy indoor air levels may be difficult to achieve in countries where biomass is in high demand for household energy needs, as in present‐day Paraguay. In Latin America, Paraguay has one of the highest percentages of population dependent on biomass as the main fuel used for cooking (49%), after Haiti (91%), Guatemala (57%), Nicaragua (54%), and Honduras (51%).^2^ In addition, excessive consumption of biomass for energy production has helped sustain progressive deforestation in the country.^1^, ^12^

The present study characterizes and models indoor air pollution related to biomass burning and low‐emission cookstoves in response to the growing need to know the national state of indoor air quality, especially in rural areas where wood and charcoal are used by the majority of households. This research was conducted in collaboration with major stakeholders involved with environmental health in Paraguay: the Pan American Health Organization (PAHO) and the Dirección General de Salud Ambiental (DIGESA). The measurements and analyses presented in this article provide a foundation for establishing a baseline that could be used in future studies, as well as in potential cookstove intervention projects.

## 3 METHODS

### 3.1 Study site

**Figure 1:**
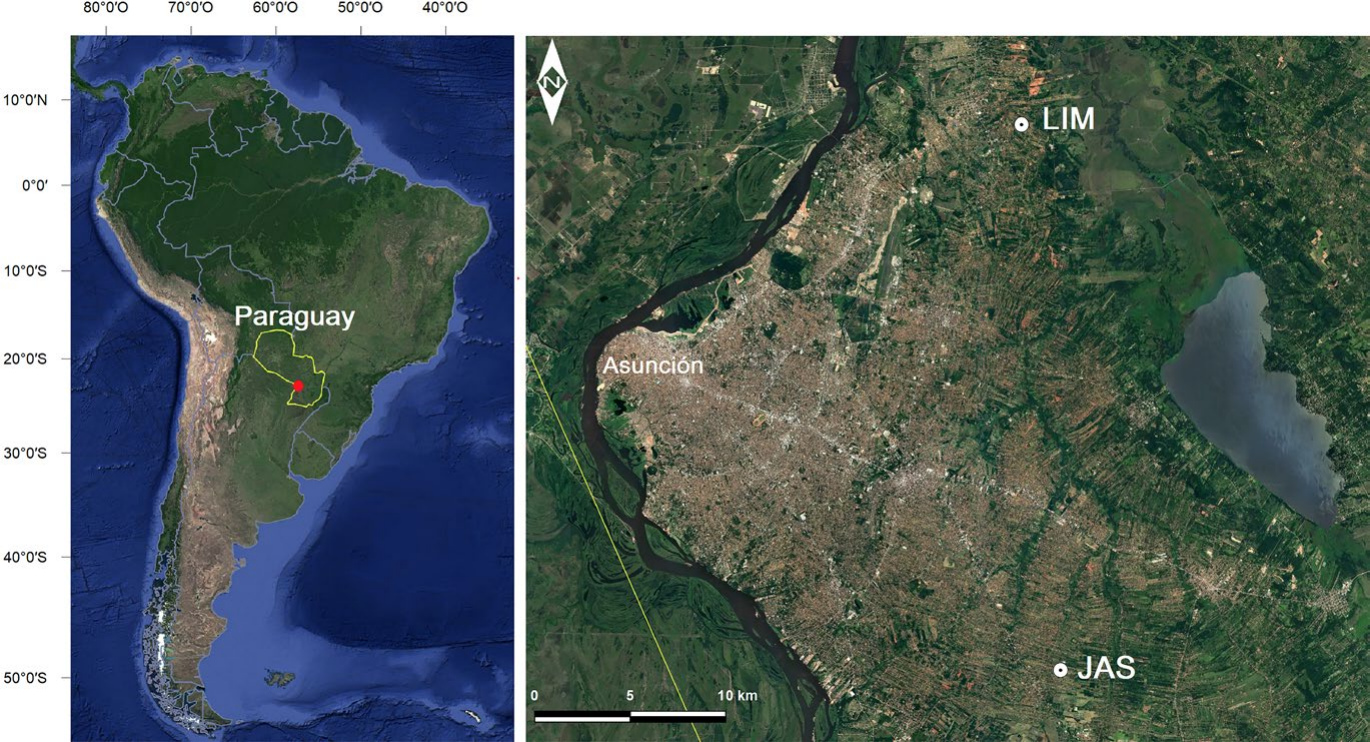
Map of the study locations. Left: Paraguay, South America (enlarged area in red circle). Right: Rural communities at JAS (Julián Augusto Saldivar) and LIM (Limpio)

The study was conducted in July 2016 (winter) in two low‐income rural communities located in the Julián Augusto Saldívar (JAS) and Limpio (LIM) districts, Central Department, Paraguay (Figure 1). Both communities are located on the outskirts of the largest national conurbation (~2 million inhabitants), about 20 km from Asunción, the country's capital.

### 3.2 Household selection

In June 2016, survey data about fuels used for cooking, heating, and lighting were collected in 238 rural households at JAS and LIM. The survey was designed and administered by PAHO based on WHO's World Health Survey. A database was created without personal identifiers but including household information, such as location and the type of fuel used for cooking (wood, charcoal, LPG, and electricity). Households were stratified according to the type of fuel used for cooking, and, in each subset, households were randomly selected to be visited each day for conducting measurements. The field team introduced the study and its measurements to the head of the family, who was invited to participate. Formal recruitment occurred after informed consent was obtained from the head of the family. Households with pregnant women or smokers were excluded.

All procedures were approved by the Committee for the Protection of Human Subject at the University of California, Berkeley (No. 2016‐02‐8451), the Ethics Committee of Research in Paraguay (CEI‐LCSP No. 42), and the Ministry of Public Health and Social Welfare (No. 73/310516).

### 3.3 Indoor air monitoring

**Figure 2:**
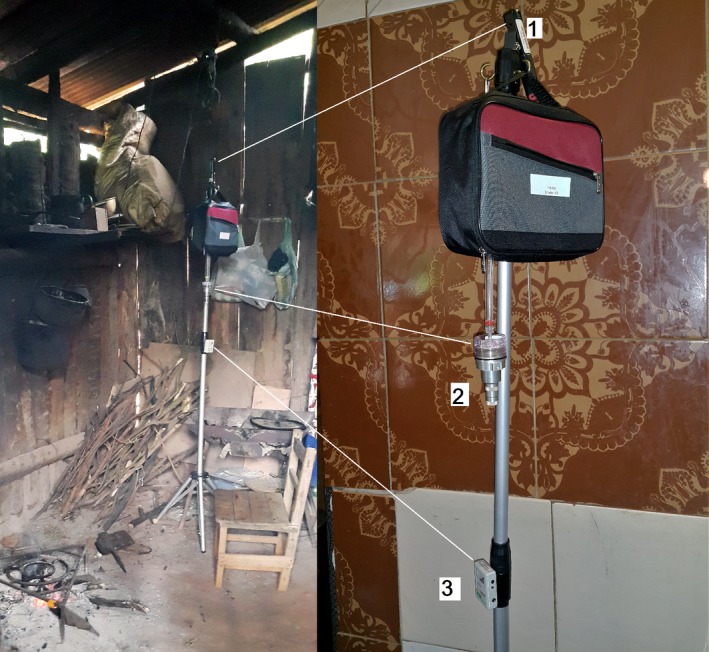
Household air pollution monitors colocated in the kitchen area. CO monitor (1), triplex cyclone for PM_2.5_ collection (2), and air temperature sensor (3)

Household air pollution was monitored in the cooking area for 24 hours. A sample deployment is shown in Figure 2. Sampling was performed on weekdays, starting one morning (8‐9 am) and ending the subsequent morning. PM_2.5_ and CO monitors were colocated approximately 1.5 m away from the cookstove and at adult breathing height (1.6 m above the floor).

PM_2.5_ was collected on a pre‐weighed PTFE (polytetrafluoroethylene) filters (Pall Corporation, NY, USA) for posterior gravimetric analysis (37 mm, 2.0 μm pore size). Filters were placed inside a three‐piece cassette (23370‐U, Sigma‐Aldrich) and backed with a drain disk (36 mm, Whatman^®^). Cassettes were coupled to a PM_2.5_ cyclone sampler (Triplex SCC1.062, Mesa Labs, USA) and connected to an air pump (AirChek XR5000, SKC, USA). The initial flowrate was adjusted to 1.5 L/min. At the beginning and end of the sampling event, the flowrate was measured using a digital flowmeter (Challenger CH100 flowmeter, Mesa Labs). To estimate the volume of air sampled (m^3^), the average of the pre‐ and post‐sampling airflow rates (m^3^/min) was multiplied by the total sampling minutes displayed on the pump screen.

For gravimetric analysis, filters were weighed before and after PM_2.5_ collection using an analytical microbalance at UC Berkeley (Mettler Toledo XP2U, USA). Filters were conditioned for 24 hours in a temperature‐ and humidity‐controlled room (23°C, 40% RH), passed between polonium‐210 metal strips to remove static, and weighed until a stable value was achieved (the last two weight values differed by 5 μg or less). The PM_2.5_ mass concentration was estimated by dividing the difference between the weight of the filter before and after the monitoring by the corresponding volume of air sampled.

Triplex cyclones were cleaned with 70% ethanol solution prior to sampling. Cassettes were checked for leaks (Field Cassette Leak Tester, SKC), capped at both ends, and transported inside hermetically sealed bags (Ziploc^®^). At the sampling location, the cassettes were uncapped and connected to the air pump using a Tygon^®^ tube and plastic Luer adapters. After sampling, the cassettes were capped, sealed with aluminum foil, and transported in cold containers until final storage at −20°C.

Field blank filters were placed in randomly selected households (n* *=* *3). Blanks were not connected to an airflow but were placed in identical cassettes and subjected to the same protocol as the filters used to collect the samples. An average increase in weight of 8.3 μg (SD 8.9) was observed; this value was subtracted from the mass collected on the sampled filters.

CO concentrations were recorded as one‐minute averages using an electrochemical sensor (EL‐USB‐CO, Lascar Electronics, UK). To ensure comparability, these sensors were intercompared by parallel measurements recorded inside a smoke chamber (Energy, Climate and Health Laboratory, UC Berkeley).

Operational variables were kept as close as possible to the expected target values. The mean (±standard deviation) of the total sampling time was 22.9 hours (±0.6), the distance from the monitors to the cookstove was 1.54 m (±0.37), and the mean flowrate at the end of sampling was 1.49 L/min (±0.05).

### 3.4 Outdoor air monitoring

In order to determine the PM_2.5_ concentration outdoors, a central location in each village was selected for installing a fixed monitoring station. The equipment was placed on the roof of households that only used electricity for cooking, approximately 2.5 m above the ground and away from direct emissions of any kind. Time‐integrated (24 hours) PM_2.5_ samples were collected on 37‐mm PTFE filters using a two‐stage impactor (4 L/min) described elsewhere.^13^ Sampled filters were subjected to the gravimetric analysis described in Section 3.5 and to X‐ray fluorescence (XRF) spectrometry to quantify concentrations of elements ranging in atomic number from 11 (Na) to 82 (Pb). The XRF spectrometry was performed with the Epsilon 5 spectrometer (Malvern Panalytical, The Netherlands) in the T.H. Chan School of Public Health, Harvard University. The chemical elements detected in outdoor PM_2.5_ samples were subjected to a principal component analysis (PCA) in order to determine the relative contribution of likely sources. In addition, one‐minute PM_2.5_ concentrations were recorded by a laser photometer (DustTrak II Aerosol Monitor 8530, TSI), which was calibrated against parallel sampling with the impactor (*R* ^2^ = 0.8). To determine the daily profile, the one‐minute PM_2.5_ concentrations were averaged over one hour. Meteorological parameters, such as hourly wind direction, wind speed, precipitation, and temperature, were obtained from the Agricultural Science Department of the National University of Asunción. The faculty operates a meteorological station located 13 and 18 km from JAS and LIM, respectively (25°20′0.04″S, 57°31′0.02″W).

### 3.5 Predictor variables

During the monitoring campaign, potential predictors of PM_2.5_ and CO concentrations were recorded as either categorical or continuous variables. A structured questionnaire was applied at both the beginning and end of the monitoring session to capture several variables at the kitchen level, as shown in Table 1. Variables included the rural community; main fuel used for cooking; the construction materials of the roof, floor, and walls; kitchen structure; occurrence of sweeping, heating, and smoking; as well as burning of incense, mosquito repellent (indoors), and garbage (outdoors). Community‐level statistics are presented in Table S1.

**Table 1:** Categorical variables captured by the questionnaire

Variable	N	Categories
Community	2	JAS LIM
Fuel	4	LPG Electricity Wood Charcoal
Kitchen structure	2	Enclosed (4 walls and a roof) Semi‐enclosed (3 walls and a roof)
Roof material	4	Ceramic (tiles) Fibrecement Metal/zinc Thatch
Wall material	4	Concrete/bricks Metal Nylon Wood
Floor material	4	Ceramic Concrete Soil Wood
Sweeping	2	Yes/No
Heating	2	Yes/No
Smoking	2	Yes/No
Mosquito coil burning	2	Yes/No
Garbage burning (outdoors)	2	Yes/No

The parameters recorded as continuous variables are shown in Table 2. Cookstove usage was monitored for 24 hours using a temperature sensor (iButton DS‐1922T, Maxim Integrated, CA, USA) as a stove use monitor (SUM).^14^, ^15^ These sensors were attached with tape to the base of cooking appliances and recorded temperature every 1 minute (T_stove_). Temperature inside the kitchen (T_air_) was recorded using a HOBO datalogger (Onset Inc, USA) colocated with the air samplers. The total time of cookstove usage was the sum of minutes in which the T_stove_ was at least 10°C above T_air_. For measuring the kitchen room volume and distance between the air samplers and the cookstove, a laser length meter was used (GLM 40, Bosch).

**Table 2:** Continuous variables assessed

Variable	Unit
Cookstove usage	Minutes
Sampler‐cookstove distance	m
Kitchen room volume	m^3^
Monitoring duration	Minutes

### 3.6 Data analysis

Descriptive statistics are presented as means, standard deviations (SD), and confidence intervals (CI) of the mean. To determine groups that were significantly different from each other, an analysis of variance (ANOVA) followed by Tukey's multiple comparison test was performed (significant at *P*‐value < 0.05).

Predictive models were created from the observed indoor concentrations and potential explanatory variables shown in Tables 1 and 2. As a preliminary step, the normality of the distribution of continuous variables was examined by visual inspection of skewness and the Shapiro‐Wilks test. For data that deviated from a normal distribution, values were natural log‐transformed. Explanatory variables were incorporated into a multiple linear regression (MLR) model using stepwise regression.

**Figure 3:**
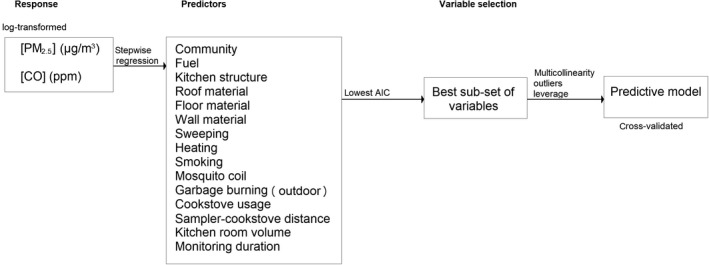
Plan of data analysis used to generate the predictive models for indoor PM_2.5_ and CO concentrations

The model with the lowest Akaike information criterion (AIC) value was selected as the regression model containing the most appropriate subset of predictor variables. Selected models were examined for multicollinearity (variance inflation factor), atypical data points (Bonferroni‐adjusted *P*‐values), and influential observations (Cook's distance). After excluding outliers, the final model was constructed on the basis of the previously identified best subset of predictor variables. The general assumptions of linear regression analysis (normality, linearity, and homogeneity of variance) were evaluated by visual inspection of residuals on the appropriate diagnostic plots. The model performance was evaluated using 10‐fold cross‐validation. A random 80% subset of the dataset was used to train the model and the remaining 20% to validate its predictions. Parameters such as adjusted *R* ^2^ and root‐mean‐square error (RMSE) were considered in the final evaluation. A detailed architecture of the data analysis is provided in Figure 3.

For statistical computing, the RStudio software (R Foundation for Statistical Computing, Vienna, Austria, R version 3.3.1) was used. The predicted *R* ^2^ in the final model was estimated using the “olsrr” package. The PCA for outdoor samples was performed with the “factoextra” and “corrplot” packages.

## 4 RESULTS

### 4.1 Cookstoves and kitchen features

The monitored households used one of four cooking methods: three‐stone open fires for burning wood, metal braziers for burning charcoal, regular LPG cookstoves, or electric hot‐plate cookers (shown in Figure S1). None of the households used more than one type of cooking method during the measurement period. Households had one of two kitchen configurations: enclosed (a kitchen inside or next to the household with a roof and four walls) and semi‐enclosed (a cooking room with a roof and three walls). Semi‐enclosed kitchens were not found in households that cooked using LPG or electricity.

Average (±SD) cookstove usage was higher in wood‐burning (8.3 ± 2.5 hours per day [h/d]) and charcoal‐burning kitchens (5.3 ± 3.0 h/d) when compared with LPG (3.4 ± 1.3 h/d) and electrical kitchens (3.9 ± 1.6 h/d). The average (±SD) kitchen volume was greater in LPG kitchens (40.1 ± 13.8 m^3^), followed by kitchens that used electricity (28.0 ± 9.4 m^3^), charcoal (26.5 ± 17.1 m^3^), and wood (20.9 ± 15.3 m^3^). The average cookstove usage was significantly different among all groups, excepting electric stoves when compared to LPG and charcoal. The only significant difference in the average kitchen volume was observed between the households using LPG and those cooking with wood and charcoal (Table S2).

### 4.2 Indoor PM_2.5_ and CO concentrations

The 24‐hour average indoor PM_2.5_ and CO concentrations observed in different kitchen and fuel settings are summarized in Table 3. The highest average (±SD) PM_2.5_ concentrations were observed in wood‐burning kitchens, specifically in the enclosed type (851 ± 656 μg/m^3^). Those kitchens using the same fuel but with a semi‐enclosed structure had a lower average PM_2.5_ concentration (681 ± 95 μg/m^3^). CO concentrations were similar between kitchen configurations.

**Table 3:** PM_2.5_ and CO concentrations in the kitchen area

Fuel and structure	N	PM_2.5_ (μg/m^3^)	CO (ppm)
Wood			
Enclosed	10	850.5 (381.2‐1320)	17.8 (5.4‐30.3)
Semi‐enclosed	18	681.2 (439.9‐922.5)	20.4 (12.4‐28.3)
Total	28	741.7 (529.8‐953.6)	19.4 (13.4‐25.5)
Charcoal			
Enclosed	10	109.1 (74.0‐144.1)	8.8 (4.0‐13.7)
Semi‐enclosed	7	104.0 (65.3‐191.5)	5.6 (0.8‐11.9)
Total	18	107.0 (71.7‐142.3)	7.6 (4.1‐11.1)
LPG			
Enclosed	24	52.3 (44.3‐60.3)	0.51 (0.19‐0.83)
Electricity			
Enclosed	10	52.0 (41.8‐62.6)	0.42 (0.14‐0.98)

Twenty‐four‐hour average and 95% CI of the mean.

In total, the enclosed and semi‐enclosed charcoal‐burning kitchens had average PM_2.5_ concentrations of 107 ± 69 μg/m^3^ and CO concentrations of 7.6 ± 6.5 ppm. Both pollutants were observed at higher concentrations in enclosed structures.

The kitchens using LPG and electricity had the lowest average concentrations of both PM_2.5_ and CO. Despite this, the average PM_2.5_ concentrations in these kitchens were 52 ± 17 μg/m^3^, higher than the values expected for an emission‐free environment.

### 4.3 Outdoor PM_2.5_ and meteorology

**Figure 4:**
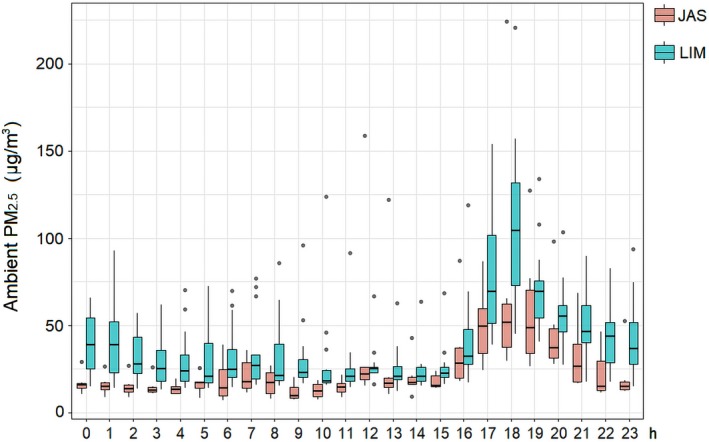
One‐hour PM_2.5_ concentrations in the outdoor environment of rural communities. The black line at the middle represents the median value for each hour, while the circles represent one‐hour concentrations outside the 25th‐75th percentiles

The 24‐hour average outdoor PM_2.5_ concentration was 27.5 μg/m^3^ (95% CI: 26.7‐28.3) in the JAS community and 41.2 μg/m^3^ (95% CI: 40.7‐41.7) in the LIM community. Figure 4 shows the variation of PM_2.5_ concentrations at different times of the day. In both villages, PM_2.5_ significantly increased between 16:00 and 20:00, suggesting the occurrence of temporary sources with large emissions. The major element found in the outdoor PM_2.5_ was potassium (K), a chemical tracer associated with biomass and agricultural burning.^16^ The mass of all the elements reported by the XRF spectrometry (Table S3) contributed 11% and 7.6% to the mass of PM_2.5_ in JAS and LIM, respectively. Decreasing in order of concentration, other predominant elements were sulfur (S), a tracer of diesel combustion, and elements of soil dust (Mg, Si, Al, Fe, Na).

The PCA result for JAS showed that elements such as S, K, Br (bromine) and Pb (lead) were strongly correlated. This finding suggests that ambient PM_2.5_ was mostly contributed by a mixture of biomass and fossil fuel combustion, in addition to street dust, due to the presence of Pb (Figure S3A). In LIM, elements associated with emissions of biomass and agricultural burning^17^ (K, Cl, Br) showed a strong correlation, as well as a considerable contribution to the total mass of PM_2.5_ (Figure S3B).

During the monitoring period, 0.10 mm^3^ of rainfall and an average (±SD) daily temperature of 18°C (±6.1) were recorded. According to the Köppen‐Geiger classification, the area is influenced by a humid subtropical climate (Cfa), which is characterized by mild winters. A daily pattern for the wind direction was observed: prevailing winds from the southwest during the night (22:00‐08:00) and northeasterly winds during the day. Based on the average speed (11 ± 8.2 m/s), the wind was categorized as a strong breeze (Beaufort scale). The wind speed reached a maximum around 11:00 in the morning (15 ± 8.2) and a minimum around 18:00 (8.2 ± 6.8), which coincided with the period of highest outdoor PM_2.5_ concentration. A bivariate linear regression indicated a negative, statistically significant association between the hourly average ambient PM_2.5_ concentration and wind speed (log‐transformed), which suggests that lower wind speeds were associated with reduced dispersion of PM_2.5_ originated within the rural environment (Table S4).

### 4.4 Indoor PM_2.5_ predictive model

**Figure 5:**
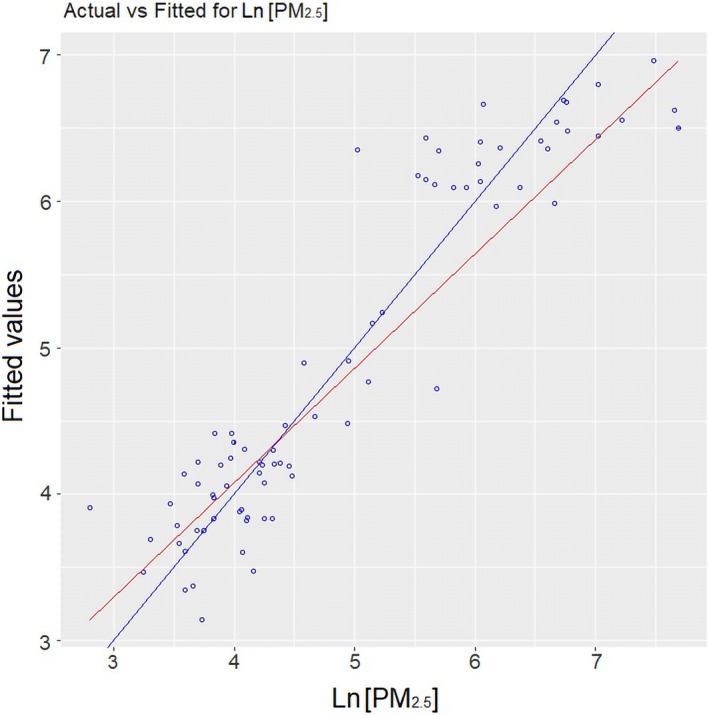
Goodness of fit of the predictive model for LnPM_2.5_. The blue line is model fit; the red line is a 1:1 line

Through the statistical procedure described in Section 4.1, a five‐predictor regression model for estimating the indoor PM_2.5_ log‐concentration (LnPM_2.5_) was derived. The model is represented by Equation (1), while its coefficients and goodness of fit are shown in Table 4 and Figure 5, respectively.

**Table 4:** Regression coefficients for Equation (1)

Variable	Coefficient (β)	Std. error	*P* value
(intercept, β_0_)	0.862	0.686	0.213
Fuel			
LPG	Reference		
Electricity	0.041	0.185	0.823
Wood	2.004	0.203	<0.001
Charcoal	0.435	0.177	0.017
Community			
JAS (Julián Augusto Saldivar)	Reference		
LIM (Limpio)	0.309	0.143	0.034
Cookstove usage			
Ln (minutes)	0.521	0.124	<0.001
Wall material			
Concrete	Reference		
Metal	−0.092	0.311	0.765
Nylon	0.159	0.194	0.414
Wood	−0.236	0.131	0.076
Garbage burning			
No	Reference		
Yes	0.276	0.124	0.029

Residual error (ε) = 0.487.

(1) $$\begin{array}{cc} & \text{Ln}({\text{PM}}_{2.5})=\\ & \;\beta_{0}+\beta_{1}*\left(\text{fuel}\right)+\beta_{2}*\left(\text{community}\right)\\ & \;+\beta_{3}*\text{Ln}\left(\text{minutes of cookstove usage}\right)\\ & \;+\beta_{4}*\left(\text{wall material}\right)+\beta_{5}*\left(\text{garbage burning}\right)+\varepsilon \end{array}$$

In general, the predictive model for LnPM_2.5_ was statistically significant (*P*‐value < 2.2^−16^) and robust (adjusted *R* ^2^ = 0.859, predicted *R* ^2^ = 0.837). About 86% of the variance in LnPM_2.5_ could be explained by predictors such as type of fuel used for cooking, the rural community, cookstove usage, and occurrence of garbage burning in nearby outdoor spaces. The most significant variables (*P*‐value < 0.001) were the fuel type, specifically wood and charcoal, as well as cookstove usage. Variables such as floor and roof construction materials, kitchen structure, and room volume were not significant in this model. Indication of garbage burning was the only variable external to kitchen emissions significantly associated with LnPM_2.5_. The occurrence of other activities such as sweeping (67.5%), smoking (3.7%), or burning mosquito repellent (3.75%) was not associated with LnPM_2.5_. The robustness of the model was supported by the high adjusted *R* ^2^ (0.807 ± 0.038) and the small RMSE (0.203 ± 0.026) obtained in the cross‐validation.

### 4.5 Indoor CO predictive model

**Figure 6:**
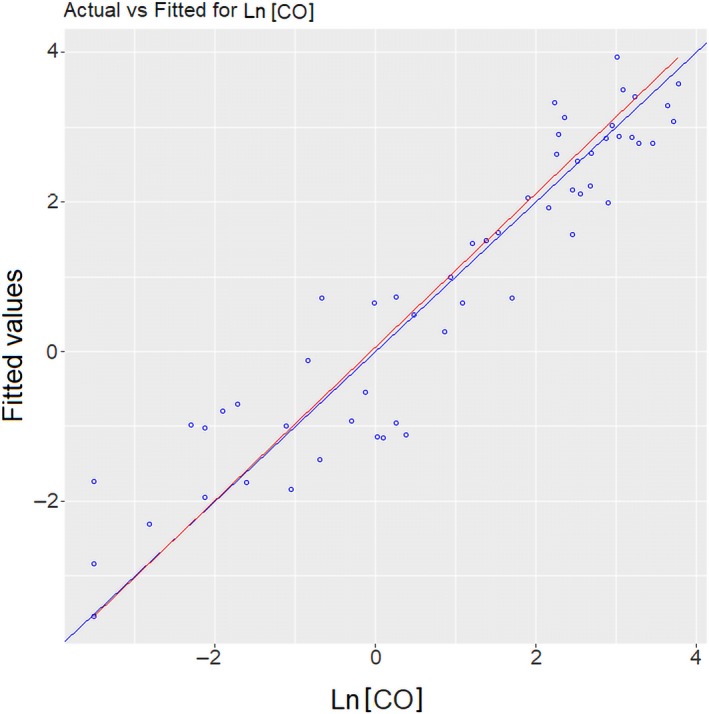
Goodness of fit of the predictive model for LnCO. The blue line is model fit; the red line is a 1:1 line

Equation (2) shows the significant (*P*‐value < 2.2^−16^) and robust (adjusted *R* ^2^ = 0.857, predicted *R* ^2^ = 0.822) five‐predictor regression model derived for the indoor CO log‐transformed concentration (LnCO). The coefficients and goodness of fit of this model are shown in Table 5 and Figure 6, respectively. The burning of wood and charcoal, indoor PM_2.5_ concentration, and cookstove usage were variables significantly associated with higher CO concentrations. Similar to the predictive model for PM_2.5_, outdoor garbage burning was the only external variable significantly associated with increased CO concentration in the kitchen (*P*‐value = 0.006). In a bivariate model (Figure S2), both pollutants also presented a significant association, although the correlation was lower (*R* ^2^ = 0.63).

**Table 5:** Regression coefficients for Equation (2)

Variable	Coefficient (β)	Std. error	*P* value
(intercept)	−8.433	1.380	<0.001
PM_2.5_			
Ln (PM_2.5 _μg/m^3^)	0.547	0.191	0.006
Fuel			
LPG	Reference		
Electricity	−0.847	0.408	0.043
Wood	2.066	0.580	<0.001
Charcoal	2.469	0.362	<0.001
Floor material			
Ceramic	Reference		
Concrete	0.854	0.522	0.108
Soil	−0.138	0.562	0.807
Wood	0.603	0.760	0.431
Cookstove usage			
Log (minutes)	0.891	0.273	0.002
Garbage burning			
No	Reference		
Yes	0.563	0.258	0.034

Residual error (ε) = 0.797.

(2) $$\begin{array}{cc} & \text{Ln}(\text{CO})=\\ & \;\beta_{0}+\beta_{1}*\text{Ln}\left({\text{PM}}_{2.5}\right)+\beta_{2}*\left(\text{fuel}\right)\\ & \;+\beta_{3}*\left(\text{floor material}\right)+\beta_{4}*\text{Ln}\left(\text{minutes of cookstove usage}\right)\\ & \;+\beta_{5}*\left(\text{garbage burning}\right)+\varepsilon \end{array}$$

The cross‐validation resulted in a relatively high adjusted *R* ^2^ (0.762 ± 0.037) and a low RMSE (0.365 ± 0.033). Even though the selected model fits well, the intercept had a significant association, which indicates the existence of other unincorporated variables that may also contribute to the variation in the CO indoor concentration.

## 5 DISCUSSION

To the best of our knowledge, indoor and outdoor air quality data have not been previously reported in the scientific literature for Paraguay. Based on the results of this study, the 24‐hour average PM_2.5_ concentrations in both indoor and outdoor environments exceeded the guidelines established by the WHO (35 μg/m^3^, Interim‐Target 1). We suggest that outdoor PM_2.5_ concentrations in the rural communities could be strongly influenced by biomass burning for cooking and waste burning.

Table 6 summarizes the results from other indoor air quality studies performed in similar rural settings. The measurements obtained in this study are comparable to values presented by studies conducted in Latin American countries, such as Guatemala,^18^, ^19^ Nicaragua,^20^ Honduras,^21^ and Mexico.^22^

**Table 6:** PM_2.5_ and CO concentrations from indoor air quality studies conducted in rural environments

Location	Year	Fuel and stove	PM_2.5_ (μg/m^3^)	CO (ppm)
Paraguay (this study)	2016	Wood (open fire)	742 ± 546 (n = 28)	19 ± 13 (n = 28)
		Charcoal (brazier)	107 ± 69 (n = 18)	7.6 ± 6.5 (n = 18)
		LPG (stove)	52 ± 19 (n = 24)	0.5 ± 0.6 (n = 24)
		Electricity (hot plate)	52 ± 15 (n = 10)	0.4 ± 0.6 (n = 10)
		Outdoor	34 (26‐41) (n = 24)	
Guatemala^18^	2006‐2007	Wood (open fire)	900 ± 700 (n = 138)	6.7 ± 5.1^a^ (n = 145)
		Wood (chimney)	340 ± 490 (n = 138)	2.4 ± 4.1^a^ (n = 163)
Guatemala^19^	2004	Wood (open fire)		11.0 ± 6.7 (n = 34)
Nicaragua^20^	2008	Wood (open fire)	1354 ± 127 (n = 115*)*	26 ± 25 (n = 124)
Honduras^21^	2005	Wood (open fire)	1002 ± 1089 (n = 27)	
		Wood (improved)	266 ± 240 (n = 23)	
		Outdoor	282 ± 313 (n = 49)	
Mexico^22^	2004‐2005	Wood (open fire)	658 ± 434 (n = 37)	
		Outdoor	59 ± 18 (n = 20)	
Peru^23^	2009	Wood (open fire)	211 (116‐305) (n = 24)	5.2 (2.8‐7.5) (n = 32)
Peru^24^	2009	Wood (open fire)		7.6 (7.1‐8.1) (n = 81)
		Natural gas		4.0 (0‐9.4) (n = 4)
Peru^25^	2008	Wood (open fire)	207 (163‐265) (n = 26)	3.6 (2.6‐4.9) (n = 25)
Nepal^28^	2006‐2007	Biomass (open fire)	638 ± 810 (n = 89)	
		LPG (stove)	101 ± 141 (n = 165)	
		Electricity (stove)	56 ± 36 (n = 54)	
Nepal^29^	2010‐2011	Wood (open fire)	1186 (710‐1920) (n = 844)	8.2 (4.6‐14.5) (n = 544)
Pakistan^30^	2005‐2006	Wood (open fire)	2740 ± 2060 (n = 51)	29 ± 16 (n = 51)
		Natural gas (stove)	380 ± 390 (n = 44)	7.5 ± 4.4 (n = 44)
China^31^	2013	LPG	59 ± 42 (n = 7)	
		Electricity	49 ± 35 (n = 41)	
		Outdoor	80 ± 49 (n = 11)	

a ppm converted from mg/m^3^ (25° C, 1013 mbar).

Mean ± SD or 95% CI.

Studies in Peru have reported wood‐burning kitchens with lower PM_2.5_ and CO concentrations.^23^, ^24^, ^25^ Different cooking behaviors could explain this difference, as the wood‐burning kitchens in Peru were estimated to be used for cooking for 3.7‐3.9 h/d,^25^ while the wood‐burning kitchens in Paraguay were used for cooking for 7 h/d. This difference may also reflect the methodologies used in each study, since the estimated cooking time was based on information recorded by temperature sensors in the present study, but it was based on activity diaries in Peru. The mean cooking duration estimated for wood‐burning kitchens in Paraguay is closer to the average values reported in Mexico (6.5 h/d)^26^ and Guatemala (6.8 h/d).^27^

As shown in Table 6, a similar magnitude of PM_2.5_ and CO concentrations has also been reported in rural households in Nepal,^28^, ^29^ Pakistan,^30^ and China.^31^ As observed in Paraguay, concentrations of PM_2.5_ higher than the values expected were recorded in households that used LPG or electricity. In densely populated areas, emissions from households using solid fuels (biomass and coal) have been indicated as the main factor responsible for increasing PM_2.5_ inside homes using clean fuels.^32^, ^33^ In our study, the large contribution of K found in ambient PM_2.5_ suggests that outdoor air quality was considerably impacted by biomass burning.

From the measurements described in this paper, a baseline for indoor air quality and two predictive models were developed for rural kitchens in Paraguay. Both models had a predictive power of over 80% and may be useful for predicting new observations of PM_2.5_ and CO concentrations in kitchens with similar configurations. Regression analysis showed that variables such as the kitchen structure and construction materials were not significant, while other factors, such as the community, cookstove usage, and the type of fuel used for cooking, were strong predictors of the indoor PM_2.5_ and CO concentrations. In the literature, analogous associations were observed in regression analysis in indoor studies performed in China^34^ and Pakistan.^30^ In the first, belonging to a specific rural community was a significant predictor in PM_2.5_ levels, while in the second, the duration of biomass burning was shown to have a statistically significant association with the increase in the same pollutant.

The significant association (*P*‐value = 0.03) between LIM/JAS community variable and the household PM_2.5_ concentrations can be explained by the different distribution of cooking with biomass among the two communities. In LIM, 61% of the population used biomass for cooking, in contrast to 47% observed in JAS (Table S1). A greater proportion of the population using LPG and electricity resulted in a lower average concentration of indoor PM_2.5_ at the community level, which was reflected in the model as a significant covariate.

The relevance of using the CO concentration as a proxy for indoor PM_2.5_ has been discussed in the literature. Some studies reported a relatively strong correlation (Pearson's *r *>* *0.8) between both pollutants,^35^, ^36^ while others found a weaker correlation,^18^, ^37^ especially at the level of personal exposure.^38^ Based on our regression analysis, we can indicate that CO was strongly and significantly associated with the variation in PM_2.5_ levels (*P*‐value = 0.006) in the kitchen area. This would support the methodology used in other studies of household air pollution, which have estimated PM_2.5_ from measurements made in CO concentrations.^18^, ^39^

Although the performance of the model was robust for both PM_2.5_ and CO, limitations such as relatively low sample size can be identified. The representativeness of the model for other populations is also an important limitation, since the community variable was a significant predictor. The models delivered by this study could be refined by incorporating a greater number of observations.

## 6 CONCLUSION

For the first time in Paraguay, indoor air quality has been evaluated for households that use different cooking fuels. The study observed that kitchens burning wood and charcoal resulted in the highest average concentrations of both PM_2.5_ and CO, exceeding by far the values recommended by the WHO as safe for health. The lowest concentrations of both pollutants were observed in kitchens that used LPG or electricity; however, these kitchens had higher‐than‐expected PM_2.5_ concentrations; this could be associated with external sources, such as burning of biomass and garbage in community spaces. Two regression models were developed to estimate indoor PM_2.5_ and CO concentrations, which have a predictive power of over 85%. Both models can be considered when designing national cookstove intervention projects, as well as in cost‐benefit analysis.
