# Supplementary material for: Monitoring and modeling of household air quality related to use of different Cookfuels in Paraguay
Source: Indoor Air. 2019 Jan 30;29(2):252–62. doi: 10.1111/ina.12513 (PMC6849814; doi:10.1111/ina.12513)
Supplement: Supplementary file 1 [file INA-29-252-s001.docx]

10.1111/ina.12513

Online supporting information for the following article published in Indoor Air, DOI: TO BE ADDED BY THE PRODUCTION EDITOR

Title: Monitoring and Modeling of Household Air Quality Related to Use of Different Cookfuels in Paraguay

Supporting Information

Monitoring and modeling of household air quality related to use of different cookfuels in Paraguay

^*1,4^Tagle Matias, ^1^Pillarisetti Ajay, ^1^Hernandez Maria Teresa, ^2^Troncoso Karin, ^2^Soares Agnes, ^2^Ricardo Torres, ^3^Galeano Aida, ^4^Oyola Pedro, ^1,5^Balmes John, ^1^Smith Kirk R.

^1^Environmental Health Sciences, School of Public Health, University of California at Berkeley, 2121 Berkeley Way, CA 94720, USA, ^2^Pan American Health Organization, 525 23rd Street, Washington, DC 20037, USA, ^3^Dirección General de Salud Ambiental, Avda. Gral. Eugenio A. Garay 152, San Lorenzo, Paraguay, ^4^Centro Mario Molina Chile, Antonio Bellet 292, Providencia, Santiago, Chile, ^5^School of Medicine, University of California, San Francisco, 1001 Potrero Ave, CA 94110, USA.

^*^*Corresponding author: mtagle@berkeley.edu*


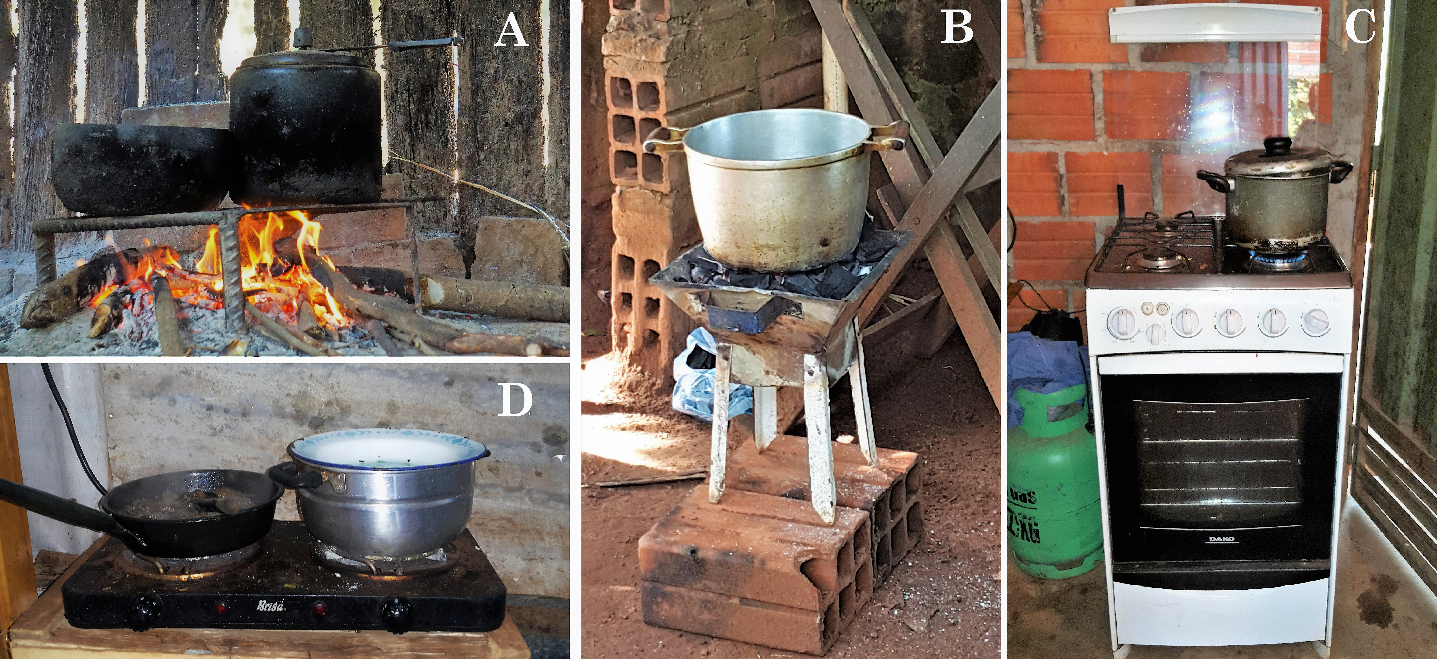


Supporting Figure 1. Cookstoves commonly found in the rural villages.

A: wood-burning open fire. B: charcoal brazier. C: LPG regular cookstove. D: double-surface electric hot plate.


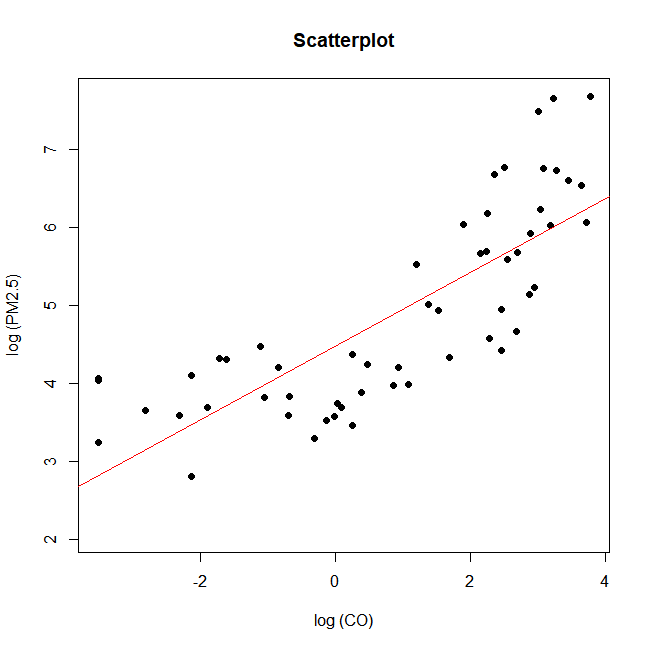


Supporting Figure 2. Correlation between log-transformed CO and PM_2.5_ indoor concentrations (R^2^ = 0.63).

Supporting Table 1. Summary statistics of categorical variables used as predictors in each rural community.

|  | JAS  n=18 | LIM  n=62 |
| --- | --- | --- |
| **Fuel**  LPG  Electricity  Wood  Charcoal | 7  3  5  3 | 17  7  23  15 |
| **Kitchen structure**  Enclosed  Semi-enclosed | 14  4 | 40  22 |
| **Roof material**  Ceramic (tiles)  Fibrecement  Metal/Zinc  Thatch | 0  10  6  2 | 17  26  18  1 |
| **Wall material**  Concrete/bricks  Metal  Nylon  Wood | 3  2  4  9 | 28  1  8  25 |
| **Floor material**  Ceramic  Concrete  Soil  Wood | 0  5  12  1 | 7  17  36  2 |
| Sweeping  Y  N | 11  7 | 45  17 |
| Heating  Y  N | 0  18 | 0  62 |
| Smoking  Y  N | 1  17 | 2  60 |
| Mosquito coil burning  Y  N | 0  18 | 3  59 |
| Garbage burning (outside)  Y  N | 5  13 | 19  43 |

Supporting Table 2. Results of Tukey's multiple comparison test for means that are significantly different from each other. Significance: *** P < 0.001, ** P < 0.01, * P < 0.05.

|  | Mean difference | 95% CI of difference | *significant?* |  |
| --- | --- | --- | --- | --- |
|  |  |  |  |  |
| *Ln (cookstove usage)*  LPG vs Wood | -0,41 | -0,56 to -0,27 | Yes | *** |
| LPG vs Charcoal | -0,18 | -0,35 to -0,014 | Yes | * |
| Wood vs Charcoal | 0,23 | 0,07 to 0,39 | Yes | ** |
| Electric vs Wood | -0,35 | -0,54 to -0,17 | Yes | *** |
| Electric vs Charcoal | -0,12 | -0,33 to 0,08 | No | ns |
| Electric vs LPG | -0,06 | -0,25 to 0,13 | No | ns |
| *Ln (kitchen room volume)* |  |  |  |  |
| LPG vs Wood | 0,35 | 0,18 to 0,53 | Yes | **** |
| LPG vs Charcoal | 0,20 | 0,004 to 0,40 | Yes | * |
| Wood vs Charcoal | -0,15 | -0,34 to 0,03 | No | ns |
| Electric vs Wood | 0,19 | -0,03 to 0,41 | No | ns |
| Electric vs Charcoal | 0,04 | -0,21 to 0,28 | No | ns |
| Electric vs LPG | 0,16 | -0,06 to 0,40 | No | ns |

Supporting Table 3. Average concentrations (μg/m^3^) of elements in outdoor PM_2.5_.

Mean and (SD).

|  | JAS | LIM |
| --- | --- | --- |
| K | 1.47 (0.79) | 1.22 (0.35) |
| S | 0.59 (0.28) | 0.75 (0.26) |
| Mg | 0.39 (0.21) | 0.37 (0.18) |
| Si | 0.10 (0.06) | 0.24 (0.13) |
| Al | 0.12 (0.08) | 0.19 (0.10) |
| Fe | 0.07 (0.03) | 0.13 (0.06) |
| Na | 0.05 (0.03) | 0.13 (0.07) |
| Cl | 0.10 (0.14) | 0.06 (0.04) |
| Ca | 0.06 (0.02) | 0.05 (0.02) |
| Zn | 0.015 (0.007) | 0.020 (0.008) |
| Ti | 0.009 (0.006) | 0.015 (0.010) |
| Br | 0.007 (0.006) | 0.011 (0.005) |
| Others* | 0.093 | 0.065 |

*sum of average concentrations of Ga, Ag, Ba, Nb, Tl, Co, Au, Ni, Hg, V, In, Y, Zr, Pd, Cd, Sn, Sb, As, Mo, Cr, Se, Sr, Mn, W, Pb, Eu, Cu, P, Cs, Rb, Ce, Br, Sc, La, Sm, Tb.


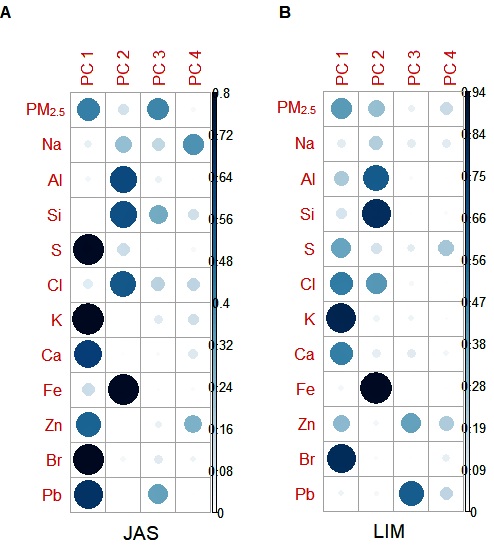


Supporting Figure 3. PCA results of ambient PM_2.5_ samples.

The correlation plot shows the degree of correlation between each element with each principal component (PC). The plot highlights the most contributing variables for each possible source (expressed in percentage). For JAS and LIM, the 4-solution result explains 88% and 83% of the variance, respectively.

Supporting Table 4. Regression parameters between one-hour average wind speed and PM_2.5_ concentration (log-transformed). ***: significance 0.001.

| Variable | Coefficient (β) | *p* value |  |
| --- | --- | --- | --- |
| (intercept) | 3.195 | < 2e-16 | *** |
| PM_2.5_  Log (μg/m^3^) | -0.285 | 2.22e-17 | *** |
